# A nomogram for predicting lung-related diseases among construction workers in Wuhan, China

**DOI:** 10.3389/fpubh.2022.1032188

**Published:** 2022-12-12

**Authors:** Xuyu Chen, Wenjun Yin, Jie Wu, Yongbin Luo, Jing Wu, Guangming Li, Jinfeng Jiang, Yong Yao, Siyu Wan, Guilin Yi, Xiaodong Tan

**Affiliations:** ^1^School of Public Health, Wuhan University, Wuhan, Hubei, China; ^2^Wuhan Prevention and Treatment Center for Occupational Diseases, Wuhan, Hubei, China

**Keywords:** nomogram, lung-related diseases, construction workers, occupational exposure, occupational health

## Abstract

**Objective:**

To develop a prediction nomogram for the risk of lung-related diseases (LRD) in construction workers.

**Methods:**

Seven hundred and fifty-two construction workers were recruited. A self- designed questionnaire was performed to collected relevant information. Chest X-ray was taken to judge builders' lung health. The potential predictors subsets of the risk of LRD were screened by the least absolute shrinkage and selection operator regression and univariate analysis, and determined by using multivariate logistic regression analysis, then were used for developing a prediction nomogram for the risk of LRD. C-index, calibration curve, receiver operating characteristic curve, decision curve analysis (DCA) and clinical impact curve analysis (CICA) were used to evaluation the identification, calibration, predictive ability and clinical effectiveness of the nomogram.

**Results:**

Five hundred and twenty-six construction workers were allocated to training group and 226 to validation group. The predictors included in the nomogram were symptoms, years of dust exposure, work in shifts and labor intensity. Our model showed good discrimination ability, with a bootstrap-corrected C index of 0.931 (95% CI = 0.906–0.956), and had well-fitted calibration curves. The area under the curve (AUC) of the nomogram were (95% CI = 0.906–0.956) and 0.945 (95% CI = 0.891–0.999) in the training and validation groups, respectively. The results of DCA and CICA indicated that the nomogram may have clinical usefulness.

**Conclusion:**

We established and validated a novel nomogram that can provide individual prediction of LRD for construction workers. This practical prediction model may help occupational physicians in decision making and design of occupational health examination.

## Introduction

Pulmonary diseases are a common and frequently-occurring disease, which have posed a serious threat to human health due to its high morbidity and mortality rate (1). In construction industry, lung diseases usually occur in the operating post with high probability of dust exposure: silica dust generated by excavator operators when digging earthwork, sawdust generated by woodworkers due to cutting wood formwork, cement dust produced by cement mixer due to operation of mixer, welding fume generated during welding by electric welders, etc. Lungs are very sensitive to dust, therefore the incidence of chronic obstructive pulmonary disease (COPD) (2), interstitial lung disease (3), pulmonary fibrosis (4), pneumoconiosis in builders may be higher than that of the general population.

China is a rapidly developing country in the construction industry. As the core force of infrastructure construction, there are plenty of people engaged in the construction industry. In 2021, the total number of construction workers in China was about 55.58 million (5). With the advancement of urbanization in China, the demand for construction workers is also increasing. High work intensity and relatively harsh working environment have led to increased opportunities for construction workers to be exposed to more occupational hazards. In addition, high mobility and unhealthy lifestyles (excessive tobacco use and alcohol consumption), were paid less attention to, which makes workers accompanied by more disease risk factors. Therefore, it is particularly vital to monitor the health level of workers.

Globally, construction workers face an increased risk of work-related ill-health and injuries (6, 7). Increased incidence of contact dermatitis (1.4%), skin tumors (1.6%), musculoskeletal diseases (MSD, 1.9%), mesothelioma (7.1%), lung cancer (5.4%), pneumoconiosis (5.5%) and other benign pleural diseases (7.1%) among UK male construction workers aged under 65 years compared with the rest of the working population (8). An American study (9) clarified that compared with white-collar workers, construction workers had increased odds of arthritis (66.2%), back problems (36.3%), work disability (36.3%), chronic lung disease (15.2%), and work-related injuries (4.2%) after controlling for possible confounders. Another study in Nepalese (10) indicated that Upper respiratory tract infection (23.4%) and injuries (16.7%) are the most common presentation among construction workers.

Exposure to ambient particulate matter pollution contributed substantially to the global burden of respiratory diseases (11). Three of the top ten global disease causes of death in 2019 were LRD, of which COPD ranked third, lower respiratory tract infection ranked fourth, and trachea, bronchus and lung cancer ranked sixth (12). Deng et al. (13) found that the burden of tracheal, bronchus, and lung cancer was the greatest in Asia, followed by high-income North America. Due to occupational exposure to various dust, pulmonary disease was also one of the most common diseases of construction workers. Many studies verified that occupational exposures were associated with incidence of LRD in construction workers (14, 15). In recent years, there are relatively few studies on construction workers in China, most of which have focus on unsafe behaviors (16–18) and MSD (19), while there are fewer studies on LRD risk and individual prediction of construction workers. At the same time, logistic regression is usually used for screening out the high risk factors of LRD, but quantitative evaluation method of the risk is rare. The nomogram model can visualize the results of logistic regression and can be directly used in the prediction of individual risk factors. Recent studies have shown that nomogram also express good predictive power in social science research without clinical characteristics, such as the prediction of adolescent bullying (20) and self-directed learning levels (21).

At present, the number and technical level of Chinese construction workers are at the leading level in the world, but the health monitoring of construction workers does not match it. Construction workers have relatively low educational background and health literacy, and they are busy with their work, little attention is paid to their own health. Coupled with their high mobility, long-term dynamic monitoring of them is a great challenge. Consequently, our study aims to develop a simple, effective and quantitative method for clinicians, especially for occupational physicians in institution of prevention and treatment center for occupational diseases, to predict the risk of LRD and provide scientific guidance for the prevention and early intervention for each construction worker.

## Methods

### Study design and subjects

A cross-sectional study was conducted from June 1 to July 31, 2022. Six construction sites were randomly selected by using cluster random sampling method in Wuhan, and all construction workers in construction sites were selected for investigation. The investigation included chest radiograph examination and questionnaire. Investigators are trained to ensure the quality of data obtained. A total of 786 construction workers initially participated in our survey. Inclusion criteria were: (1) age >18 years, (2) worked in construction industry for at least 1 year, (3) participants at work during the investigation, (4) completed the questionnaire independently after interpretation by the investigator. Exclusion criteria was: (1) chest X-ray was not performed or information of questionnaire was incomplete. Finally, 34 of these participants were excluded.

### Instrument

A self-designed questionnaire was used to collect the following information: (1) basic personal information (included name, gender, age, nation, height, weight, marital status, educational background, monthly income), (2) lifestyle information (included active smoking, passive smoking, years of active and passive smoking exposure, alcohol drinking and physical exercise habit), (3) occupational related information (included occupational type, years of exposure to dust, working hours per week, work in shifts, dust mask wearing behavior, main working posture, continuous work or not, work load), (4) clinical symptoms of lung (such as chest tightness, chest pain, shortness of breath, cough, etc.) and other chronic disease history. Body mass index (BMI) is calculated by dividing weight (kg) by the square of height (meters). The labor intensity of workers included four degrees and was assessed by three questions based on “Experimental Course for the Detection of Occupational Hazard Factors” (22): “what is your main working posture,” “is it continuous work” and “how many kilograms do you work with” (Supplementary material 1).

### Operational definitions

(1) LRD: ➀ People who have abnormal lung images detected by chest radiograph examination and were diagnosed by doctors, abnormal imaging changes include but are not limited to pneumonia, nodules, interstitial alterations, emphysema, lung tuberculosis and tumors. ➁ Self-report by participants. (2) Active smoking: smoke at least 1 cigarette a day for more than half a year (23). (3) Passive smoking. Non-smokers inhale tobacco smoke for more than 15 min on an average day or more per week (24). (4) Alcohol drinking: drink alcohol at least once a week for at least 6 months. (5) Physical exercise habit: regular physical activity of more than 20 min each time for physical exercise. (6) Symptoms: clinical symptoms relate to pulmonary diseases, encompassing chest tightness, chest pain, shortness of breath, cough, expectoration, dyspnea. (7) Work in shifts: at least last for 3 months. The definition of shift work is the arrangements of work time are outside of conventional daytime hours, which includes fixed early morning, evening and night work, as well as rotating two or three shift work (25).

### Statistical analysis

Establishing a database by double entry method through EpiData version 3.1. QQ-plot was used for assessing normality of continuous variables. Normal continuous variables were expressed with means and standard deviations, and were analyzed using *t*-test for two group comparisons. Whereas non-normal continuous variables were expressed with medians and interquartile range (IQR), and were analyzed using *Mann-Whitney U*-test for two group comparisons. Pearson's Chi-square test was performed to analyses categorical data.

The data was randomly assigned to the training group (*n* = 526) and the validation group (*n* = 226) in a ratio of 7:3. Construction of model was built by using training set and evaluation of model was assessed by using validation set. Before the implementation of least absolute shrinkage and selection operator (LASSO) regression, continuous data were standardized, and multicategorical variables were processed with dummy variables. Identification of the optimal penalization coefficient (λ) in the LASSO model was achieved by 10-fold cross-validation and the minimum criterion (26). The potential predictors subsets of risk of LRD were screened by LASSO regression and univariate analysis, and determined by using multivariate logistic regression analysis (backward: LR), then were used for developing a prediction nomogram for the occurrence of LRD. Bootstrap method (1,000 bootstrap resampling) was performed for evaluating internal validation. The discrimination of the nomogram was determined by calculating the average consistency index (C-index). Calibration curve was used to assess the calibration of the nomogram. Area under the curve (AUC) in receiver operating characteristic (ROC) curve analyses was used to evaluate the predictive ability. We also performed a decision curve analyses (DCA) to determine the suitability of our established nomogram for clinical application by estimating the net benefits at different threshold probability. Clinical impact curve analyses (CICA) was performed to predict improved probability stratification for a population size as 1,000. Statistical analysis was performed using SPSS 26 (IBM, Chicago, IL, USA) and R version 4.1.2. A *p*-value of < 0.05 was considered statistically significant.

### Ethic statements

Ethical approval was obtained from Wuhan Prevention and Treatment Center for Occupational Disease Ethics Committee (2022- WZF02). All participants provided informed written consent.

## Results

### Characteristics of construction workers

The characteristics of participants in training group and validation group were presented in Table 1. Finally, 752 participants were included (646 males, mean age was 47.13 ± 11.03 years), of whom 92 were patients with LRD. Five hundred and twenty-six workers were allocated to training group and 226 to validation group, and there was no statistical difference between the two groups in each variable except for years of dust exposure.

**Table 1:** Descriptive and other characteristics of participants in training and validation group.

**Variables**	**All**	**Training group**				**Validation group**				***P* ^#^**
		**Total**	**Without disease (*n* = 456)**	**With disease (*n* = 70)**	***P* ^$^**	**Total**	**Without disease (*n* = 204)**	**With disease (*n* = 22)**	***P* ^$^**	
Age (years, mean ± SD)	47.13 ± 11.03	46.95 ± 11.19	46.09 ± 11.36	52.53 ± 8.09	< 0.001***	47.54 ± 10.64	47.20 ± 10.70	50.77 ± 9.73	0.135	0.497
**Age (%)**										
≤ 40	198 (26.33)	136 (25.86)	131 (28.73)	5 (7.14)	< 0.001***	62 (27.43)	59 (28.92)	3 (13.64)	0.377	0.855
41–50	185 (24.60)	134 (25.48)	118 (25.88)	16 (22.86)		51 (22.57)	44 (21.57)	7 (31.82)		
51–60	325 (43.22)	225 (42.78)	183 (40.13)	42 (60.00)		100 (44.25)	90 (44.12)	10 (45.45)		
≥61	44 (5.85)	31 (5.89)	24 (5.26)	7 (10.00)		13 (5.75)	11 (5.39)	2 (9.09)		
**Gender (%)**										
Male	646 (85.90)	450 (85.55)	384 (84.21)	66 (94.29)	0.026*	196 (86.73)	175 (85.78)	21 (95.45)	0.049*	0.671
Female	106 (14.10)	76 (14.45)	72 (15.79)	4 (5.71)		30 (13.27)	29 (14.22)	1 (4.55)		
**Ethnicity (%)**										
Han	721 (95.88)	502 (95.44)	435 (95.39)	67 (95.71)	0.905	219 (96.90)	198 (97.06)	21 (95.45)	0.377	0.354
Other	31 (4.12)	24 (4.56)	21 (4.61)	3 (4.29)		7 (3.10)	6 (2.94)	1 (4.55)		
**Educational background (%)**										
Primary school and below	201 (26.73)	140 (26.62)	115 (25.22)	25 (35.71)	0.055	61 (26.99)	51 (25.00)	10 (45.45)	0.067	0.470
Middle school	341 (45.35)	231 (43.92)	199 (43.64)	32 (45.71)		110 (48.67)	99 (48.53)	11 (50.00)		
High school or technical secondary school	132 (17.55)	99 (18.82)	88 (19.30)	11 (15.71)		33 (14.60)	32 (15.69)	1 (4.55)		
Junior college or above	78 (10.37)	56 (10.65)	54 (11.84)	2 (2.86)		22 (9.73)	22 (10.78)	0		
**Martial status (%)**										
Married	655 (87.10)	455 (86.50)	388 (85.09)	67 (95.71)	0.051	200 (88.50)	182 (89.22)	18 (81.82)	0.544	0.702
Unmarried	77 (10.24)	56 (10.65)	54 (11.84)	2 (2.86)		21 (9.29)	18 (8.82)	3 (13.63)		
Divorced or widowed	20 (2.66)	15 (2.85)	14 (3.07)	1 (1.43)		5 (2.21)	4 (1.96)	1 (4.55)		
**Monthly income (Yuan, %)**										
≤ 2,500	28 (3.72)	19 (3.61)	15 (3.29)	4 (5.71)	0.500	10 (4.42)	8 (3.92)	2 (9.09)	0.484	2.181
2,501–5,000	269 (35.77)	180 (34.22)	152 (33.33)	28 (40.00)		88 (38.94)	77 (37.75)	11 (50.00)		
5,001–7,500	225 (29.92)	160 (30.42)	140 (30.70)	20 (28.57)		65 (28.76)	60 (29.41)	5 (22.73)		
7,501–10,000	204 (27.13)	148 (28.14)	131(28.73)	17 (24.29)		56 (24.78)	52 (25.49)	4 (18.18)		
>10,000	26 (3.46)	19 (3.61)	18 (3.95)	1 (1.43)		7 (3.10)	7 (3.43)	0		
BMI (kg/m^2^, mean ± SD)	24.35 ± 3.58	24.37 ± 3.59	24.45 ± 3.68	23.82 ± 2.89	0.167	24.31 ± 3.55	24.44 ± 3.60	23.08 ± 2.84	0.090	0.821
**BMI (%)**										
< 18.5	19 (2.53)	14 (2.66)	12 (2.63)	2 (2.86)	0.158	5 (2.21)	5 (2.45)	0	0.049*	0.294
18.5–23.9	361 (48.01)	250 (47.53)	214 (46.93)	36 (51.43)		111 (49.12)	94 (46.08)	17 (77.27)		
24.0–27.9	273 (36.30)	185 (35.17)	157 (34.43)	28 (40.00)		88 (38.94)	84 (41.18)	4 (18.18)		
≥28.0	99 (13.16)	77 (14.64)	73 (16.01)	4 (5.71)		22 (9.73)	21 (10.29)	1 (4.55)		
**Active smoking (%)**										
No	380 (50.53)	265 (50.38)	239 (52.41)	26 (37.14)	0.017*	115 (50.88)	107 (52.45)	8 (36.36)	0.152	0.899
Yes	372 (49.47)	261 (49.62)	217 (47.59)	44 (62.86)		111 (49.12)	97 (47.55)	14 (63.64)		
**Passive smoking (%)**										
No	344 (45.74)	241 (45.82)	214 (46.93)	27 (38.57)	0.191	103 (45.58)	93 (45.59)	10 (45.45)	0.990	0.951
Yes	408 (54.26)	285 (54.18)	242 (53.07)	43 (61.43)		123 (54.42)	111 (54.41)	12 (54.55)		
**Alcohol drinking (%)**										
No	423 (56.25)	303 (57.60)	267 (58.55)	36 (51.43)	0.261	120 (53.10)	109 (53.43)	11 (50.00)	0.759	0.253
Yes	329 (43.75)	223 (42.40)	189 (41.45)	34 (48.57)		106 (46.90)	95 (46.57)	11 (50.00)		
**Physical exercise (%)**										
No	628 (83.51)	435 (82.70)	372 (81.58)	63 (90.00)	0.083	193 (85.40)	172 (84.31)	21 (95.45)	0.160	0.361
Yes	124 (16.49)	91 (17.30)	84 (18.42)	7 (10.00)		33 (14.60)	32 (15.69)	1 (4.55)		
**Symptoms (%)**										
No	581 (77.26)	405 (77.00)	387 (84.87)	18 (25.71)	< 0.001***	176 (77.88)	172 (84.31)	4 (18.18)	< 0.001***	0.792
Yes	171 (22.74)	121 (23.00)	69 (15.13)	52 (74.29)		50 (22.12)	32 (15.69)	18 (81.82)		
**Complicated with chronic diseases (%)**										
No	556 (73.94)	396 (75.29)	349 (76.54)	47 (67.14)	0.090	160 (70.80)	143 (70.10)	17 (77.27)	0.482	0.199
Yes	196 (26.06)	130 (24.71)	107 (23.46)	23 (32.86)		66 (29.20)	61 (29.90)	5 (22.73)		
**Dust mask wearing (%)**										
No	670 (89.10)	473 (89.92)	409 (89.69)	64 (91.43)	0.653	197 (87.17)	180 (88.24)	17 (77.27)	0.144	0.266
Yes	82 (10.90)	53 (10.08)	47 (10.31)	6 (8.57)		29 (12.83)	24 (11.76)	5 (22.73)		
Years of exposure to dust (median, (IQR))	4.00 (1.00–14.00)	3.00 (1.00–12.00)	2.00 (1.00–10.00)	20.00 (11.75–32.25)	< 0.001***	7.00 (1.00–17.25)	5.00 (1.00–13.00)	25.00 (19.00–31.25)	< 0.001***	0.019*
Working hours (hours/week, mean ± SD)	61.13 ± 13.13	61.17 ± 13.12	60.93 ± 13.15	62.73 ± 12.93	0.287	61.04 ± 13.18	60.72 ± 12.06	63.96 ± 21.05	0.275	0.897
**Work in shifts (%)**										
No	626 (83.24)	435 (82.70)	384 (84.21)	51 (72.86)	0.019*	191 (84.51)	175 (85.78)	16 (72.73)	0.018*	0.541
Yes	126 (16.76)	91 (17.30)	72 (15.79)	19 (27.14)		35 (15.49)	29 (14.22)	6 (27.27)		
**Labor intensity (%)**										
Light	305 (40.56)	216 (41.06)	207 (45.39)	9 (12.86)	< 0.001***	89 (39.38)	88 (43.14)	1 (4.55)	< 0.001***	0.530
Moderate	246 (32.71)	175 (33.27)	144 (31.58)	31 (44.29)		71 (31.42)	64 (31.37)	7 (31.82)		
Heavy	93 (12.37)	66 (12.55)	51 (11.18)	15 (21.43)		27 (11.95)	21 (10.29)	6 (27.27)		
Extremely heavy	108 (14.36)	69 (13.12)	54 (11.84)	15 (21.43)		39 (17.26)	31 (15.20)	8 (36.36)		
**Occupational type (%)**										
Bricklayer	72 (9.57)	44 (8.37)	37 (8.11)	7 (10.00)	0.108	28 (12.39)	20 (9.80)	8 (36.36)	0.009**	0.607
Carpenters	131 (17.42)	89 (16.92)	74 (16.23)	15 (21.43)		42 (18.58)	38 (18.63)	4 (18.18)		
Mechanical equipment operator/driver	67 (8.91)	50 (9.51)	44 (9.65)	6 (8.57)		17 (7.52)	17 (8.33)	0		
Steel rebar worker	59 (7.85)	40 (7.60)	37 (8.11)	3 (4.29)		19 (8.41)	19 (9.31)	0		
Scaffolder	54 (7.18)	40 (7.60)	36 (7.89)	4 (5.71)		14 (6.19)	14 (6.86)	0		
Handyman	82 (10.90)	58 (11.03)	44 (9.65)	14 (20.00)		24 (10.62)	21 (10.29)	3 (13.64)		
Others	287 (38.16)	205 (38.97)	184 (40.35)	21 (30.00)		82 (36.28)	75 (36.77)	7 (31.82)		
Total	752 (100)	—	456 (100)	70 (100)	—	—	204 (100)	22 (100)	—	—

# Represents *p*-value for group comparison of training group and validation group.

$ Represents *p*-value for group comparison of participants with and without lung-related diseases in training group or validation group. *Represents P < 0.05; **Represents P < 0.01; ***Represents P < 0.001.

In the training group, 70 (13.31%) participants suffered from LRD, 450 (85.55%) were males, 502 (95.44%) were Han Chinese, educational background of 231 (43.92%) were middle school. 455 (86.50%) were married, monthly income of majority workers (34.22%) was 2,000–5,000 yuan. Average BMI was 24.37 ± 3.59 kg/m^2^. The number of active smokers, passive smokers, alcohol drinkers and exercisers were 261 (49.62%), 285 (54.18%), 223 (42.40%) and 91 (17.30%), respectively. 121 (23.00%) construction workers showed lung-related clinical symptom and 130 (24.71%) said they suffered from other chronic diseases. Only 53 (10.08%) people have dust mask wearing behavior on work. The years of dust exposure ranged from 0 to 42 years, with a median and IQR of 3.00 (1.00–12.00) years. Mean working hours per week were 61.17 ± 13.12 h. People with shift work were < 18%. More than half of them had a moderate or higher labor intensity. 89 (16.92%) participants were carpenters. The same characteristics of validation group were also shown in the Table 1.

### Variables screening based on LASSO regression

**Figure 1:**
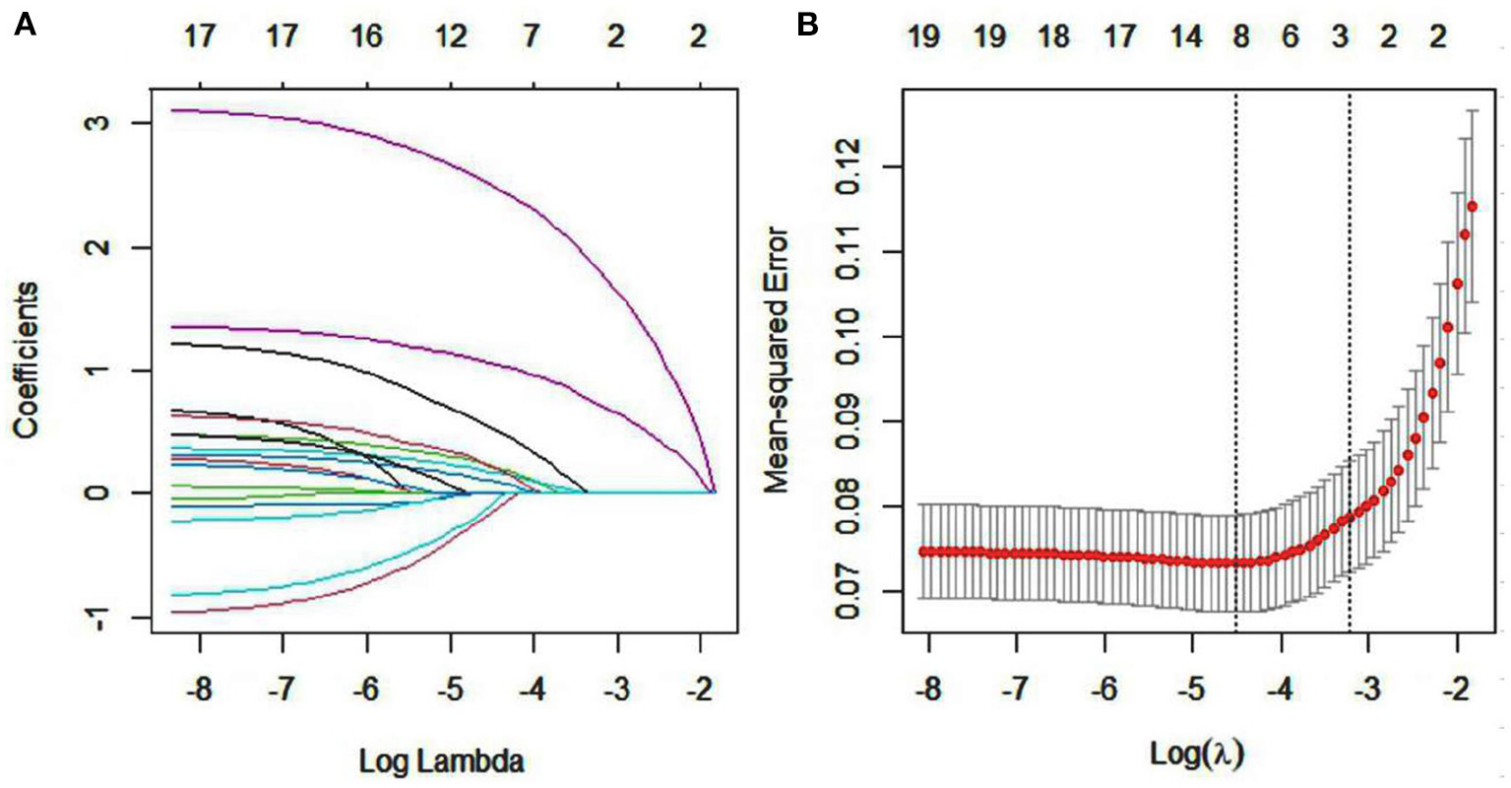
Factor selection using the LASSO logistic regression. **(A)** LASSO coefficient profile plot (left), **(B)** Cross validation plot for the penalty term.

Taking the risk of LRD as the dependent variable and the remaining variables as independent variables, a LASSO regression model was established using the training set. Figure 1A displayed the change of the regression coefficient of each independent variable under different λ. With the increase of λ, the compression degree of the model was greater, the number of independent variables entering the model was reduced, and the function of selecting main variables of the model became stronger. As shown in Figure 1B, the left vertical line represents the mean-squared error (λ.min), and the right vertical line represents the cross-validation mean-squared error with in 1 standard error of the minimum (λ.lse). In our study, λ.min was selected as the optimal value, λ.min = 0.0109. Univariate analysis showed that active smoking, dust mask wearing and occupational type were excluded. As a result, 5 variables with non-zero coefficients were selected, including age, symptoms, years of exposure to dust, work in shifts, labor intensity. The sample size met the requirements of events per variable (EPV) ≥10 (27, 28).

### Binary logistic regression and nomogram development

The remain 5 variables were subsequently filtered in the multivariate logistic regression model with a stepwise strategy. As shown in Table 2, multivariate logistic regression demonstrated that the risk of lung related diseases increased by 1.113 times (95% CI = 1.081–1.146) for each year of dust exposure. Construction workers with work in shifts (OR = 3.559, 95% CI = 1.523–8.320) and with pulmonary clinical symptoms (OR = 17.588, 95% CI = 8.268–37.413) was more likely to develop LRD. Moreover, workers with heavy labor intensity were 4.766 (95% CI = 1.589–14.295) times higher than those with light intensity.

**Table 2:** Multivariate analysis of the influencing factors of LRD in construction workers.

**Variables**	**B**	**SE**	**Wald**	***P***	**OR (95% CI)**
Years for exposure to dust	0.107	0.015	51.623	< 0.001^**^	1.113 (1.081–1.146)
Work in shifts (ref. no)	1.270	0.433	8.586	0.003^**^	3.559 (1.523–8.320)
**Labor intensity (ref. light)**					
Moderate	0.940	0.486	3.739	0.053	2.560 (0.987–6.637)
Heavy	1.561	0.560	7.763	0.005^**^	4.766 (1.589–14.295)
Extremely heavy	0.594	0.600	0.979	0.322	1.811 (0.559–5.868)
Symptoms (ref.no)	2.867	0.385	55.426	< 0.001^**^	17.588 (8.268–37.413)
Constant	−5.629	0.580	94.325	< 0.001^**^	

** Represents P < 0.01.

### Performance of the nomogram model

**Figure 2:**
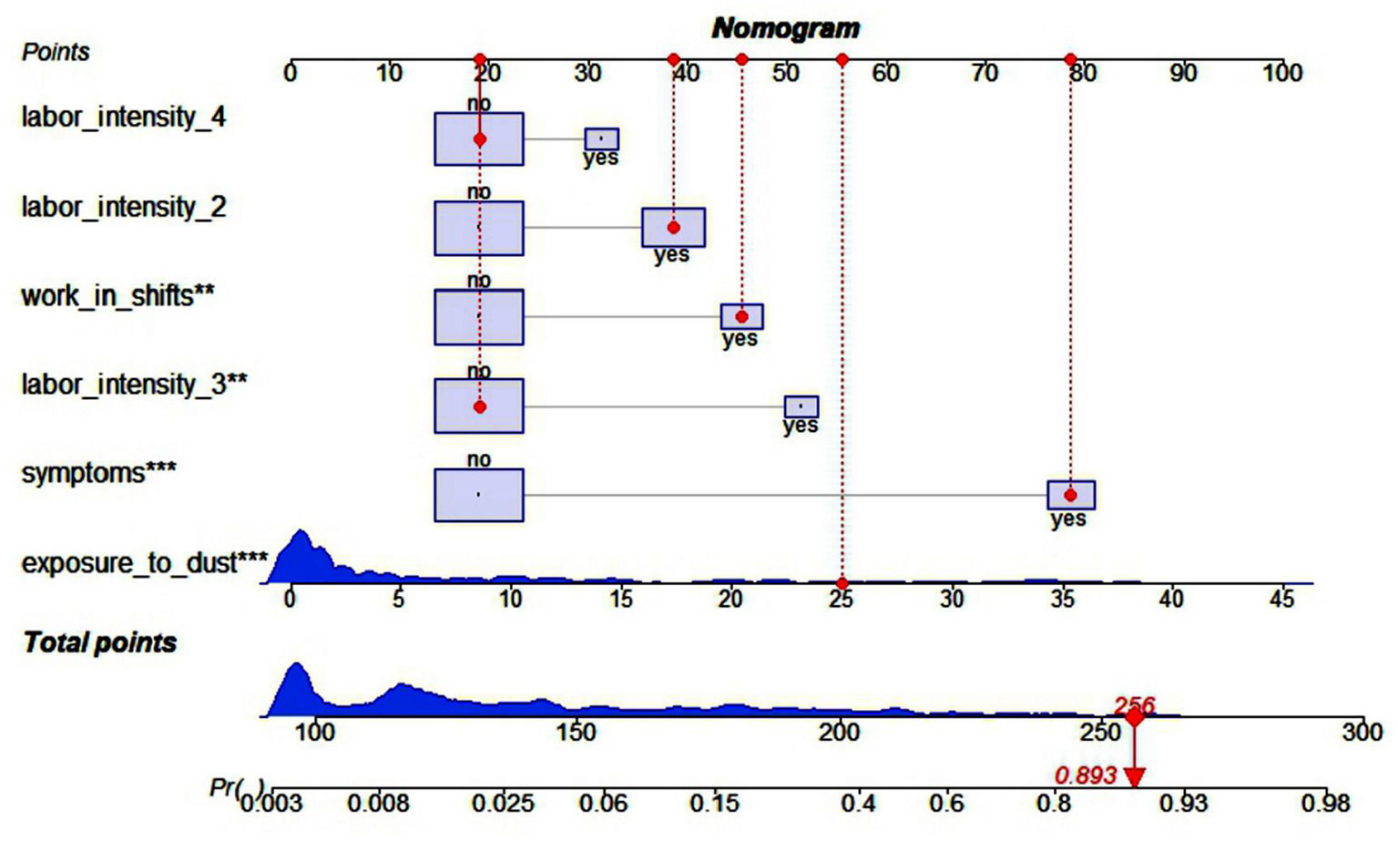
Nomogram to predict probability of the risk of LRD in construction workers. The distribution of categorical variables is displayed by lilac boxes, and distribution of continuous variables is displayed by blue density plots, the red dots represents one patient's points as an example (Observation = 111). **Represents *P* < 0.01, ***Represents *P* < 0.001.

Based on the results of logistic regression analyses, we further constructed a nomogram by combining variables including work in shifts, years to exposure to dust, labor intensity, symptoms (Figure 2). A quantitative method was made accessible for clinicians to predict the probability of LRD in each construction worker. Each worker was given a point for each predictable parameter, the higher points signified that the higher possibility of workers suffering from LRD. Then we randomly selected an observation as an example (observation 111), the situation of this object was shown by the red dot in Figure 2, the total score was 256 points, and the probability of LRD was 0.893, thus some interventions was needed to reduce the risk of LRD.

**Figure 3:**
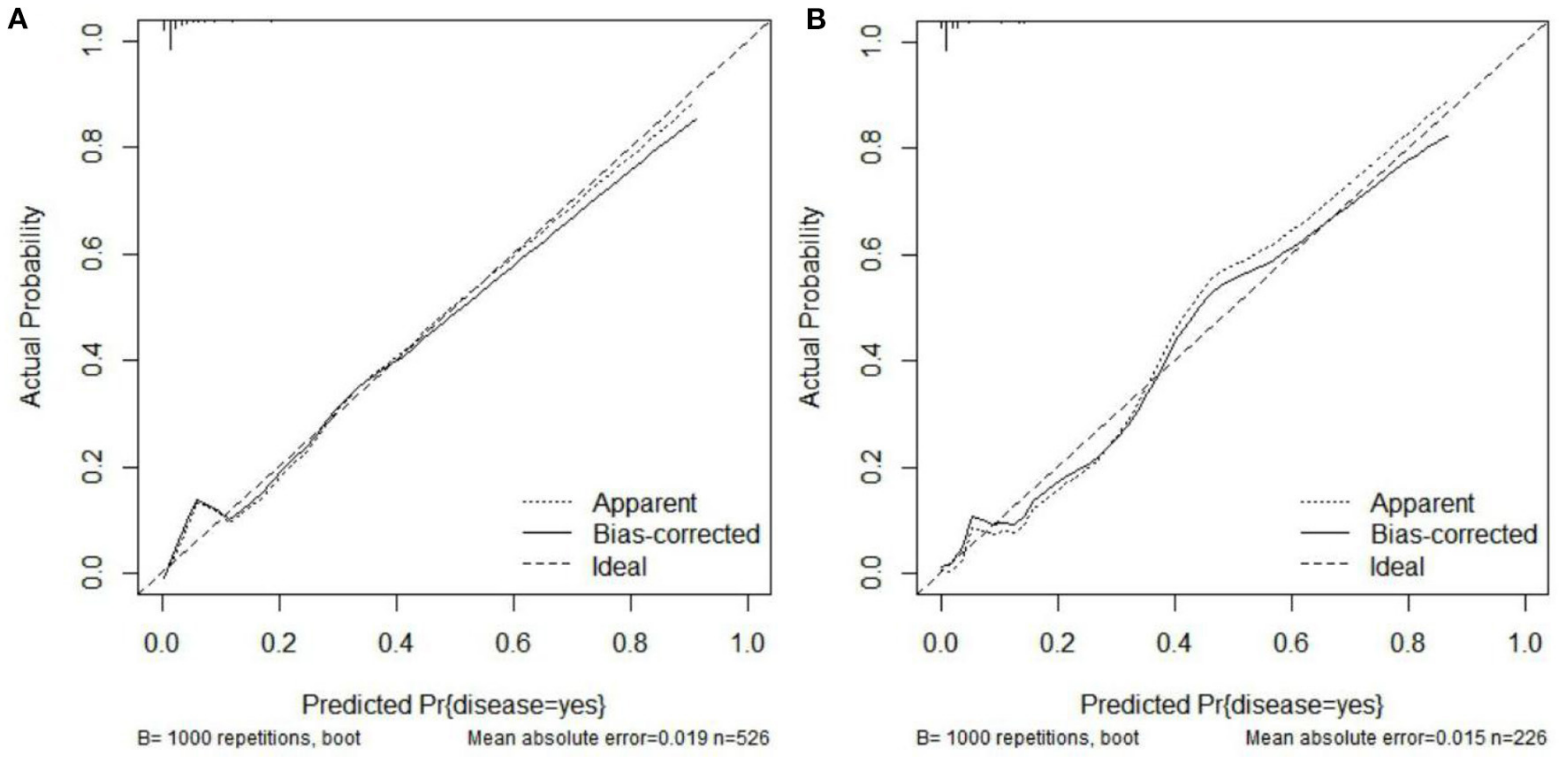
Internal consistency calibration plot of the nomogram model. **(A)** Calibration curve for the training group. **(B)** Calibration curve for the validation group.

**Figure 4:**
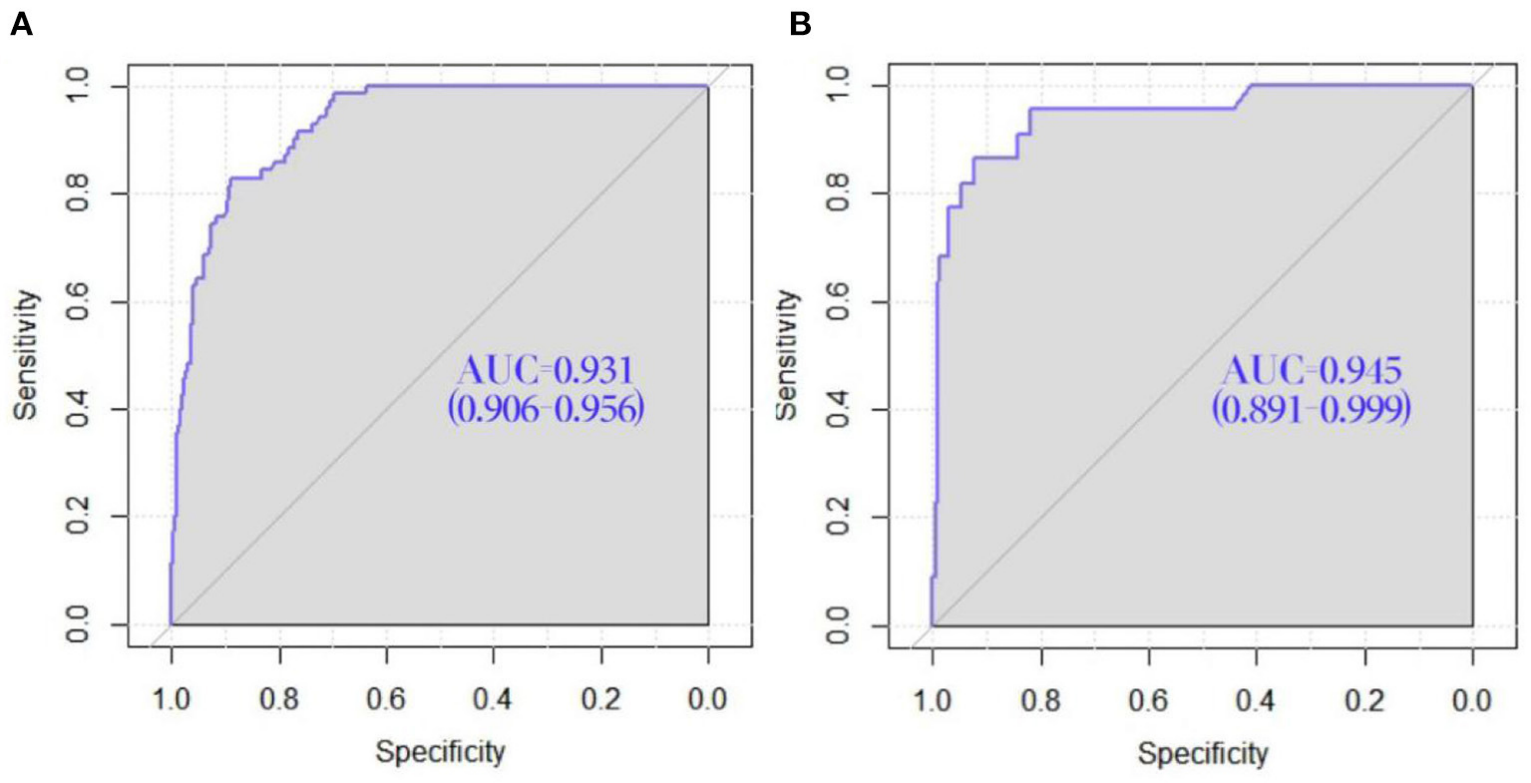
ROC curve of the nomogram for the prediction of the risk of LRD in construction workers. **(A)** ROC curve for the training group. **(B)** ROC curve for the validation group.

**Figure 5:**
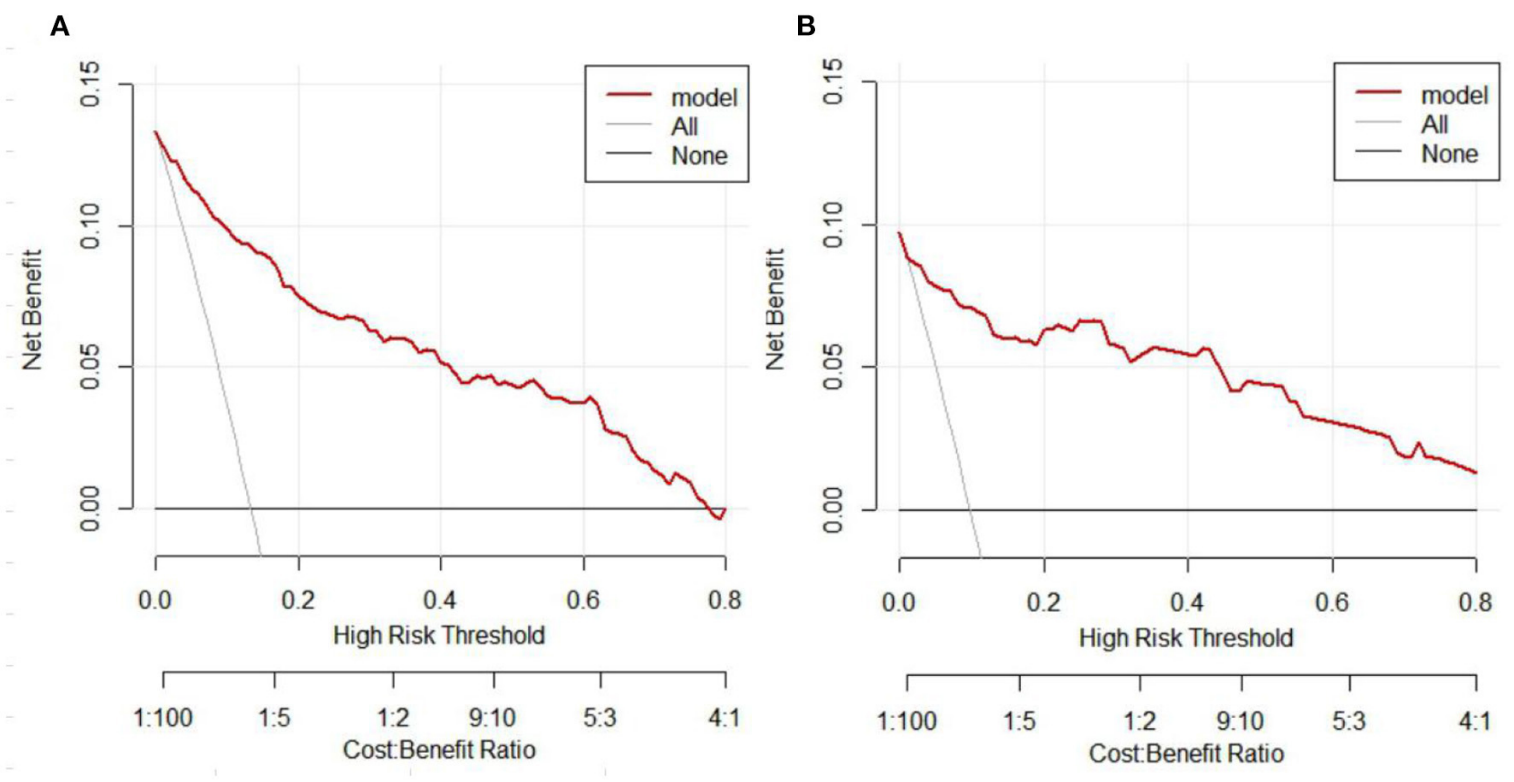
Decision curve analysis (DCA) of the nomogram. **(A)** DCA of the nomogram for the training group. **(B)** DCA of the nomogram for the validation group. The y axis represents the net benefit and the x axis represents the high risk thresholds that we chosen here range from 0 to 0.8. The black line represents the assumption of none have LRD, the gray line represents the assumption of all people have LRD.

**Figure 6:**
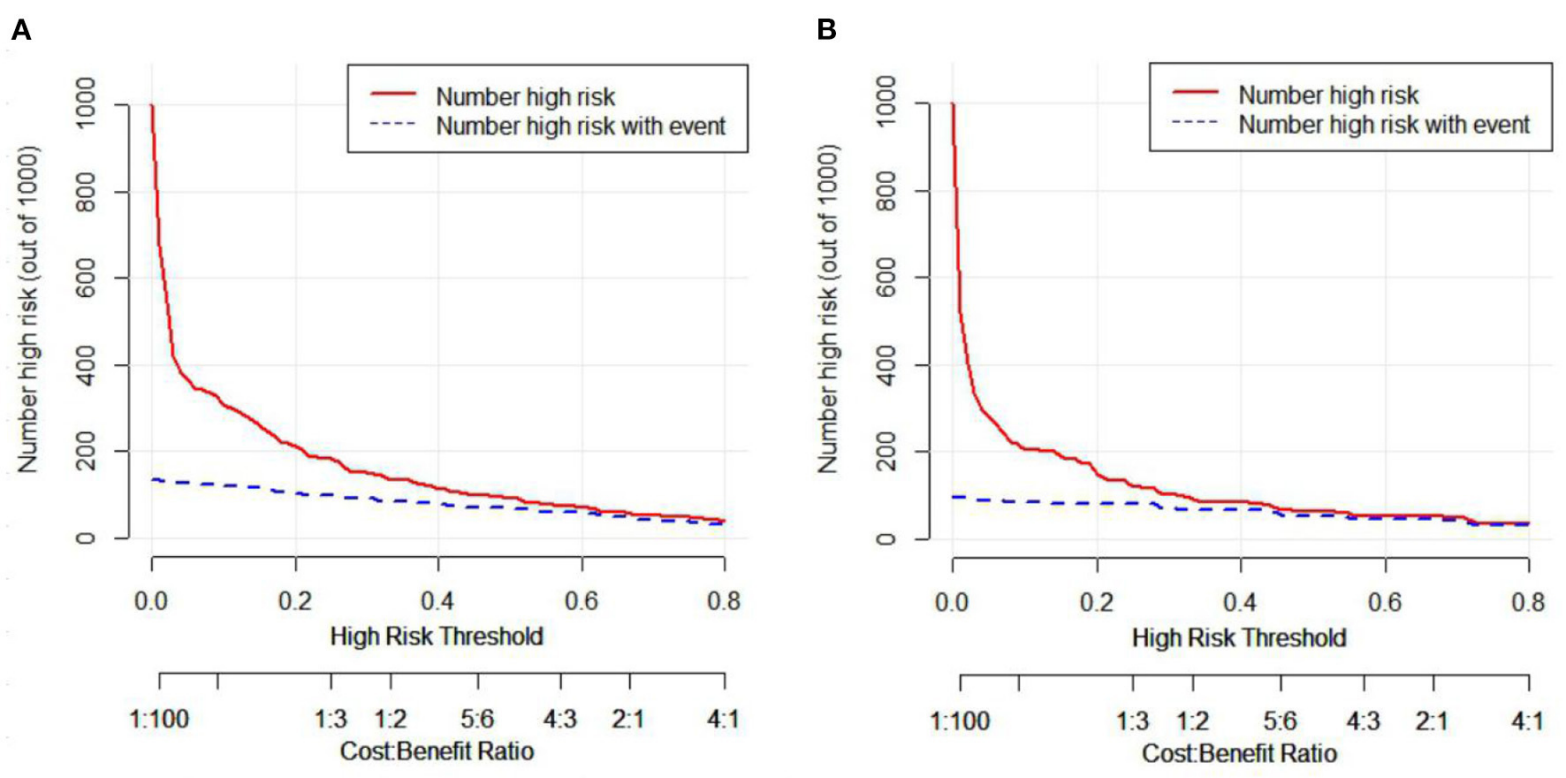
Clinical impact curve analyses (CICA) of nomogram. **(A)** CICA of the nomogram for the training group. **(B)** CICA of the nomogram for the validation group. The red curve represents the number of people who are classified as positive (high risk) by the nomogram model under each threshold probability, the blue curve is the number of people who are truly positive under each threshold probability.

The C-index of the nomogram was 0.931 (95% CI = 0.906–0.956), indicated that the model was sufficiently accurate. In training group and validation group, the calibration plots demonstrated an excellent correlation between observed and predicted LRD (Figure 3). The results of ROC curve analyses were displayed in Figure 4. The AUC was 0.931 (95% CI = 0.906–0.956) for training group and 0.945 (95% CI = 0.891–0.999) for validation group, signified the predictive accuracy of nomogram model was acceptable. In addition, DCA showed that making use of the nomogram for predicting the probability of LRD would gain more net benefit if the threshold probability was < 0.80 in validation group, which indicated a great potential for clinical utilization (Figure 5). Stratification of the LRD probability for 1,000 samples was predicted on the clinical impact curve (Figure 6). When the threshold probability was higher than 0.20, the predictive number of LRD was close to the actual number of positive cases.

## Discussion

Our study found that 92 (12.23%) construction workers had abnormal imaging change, of which 87 (94.56%) expressed relevant symptoms, which should be paid attention to. Pulmonary diseases have already become one of the major health problems for construction workers. It directly affects lung function, causing workers to suffer from breathing difficulties, hypoxia and other symptoms, and even develops into pneumoconiosis, lung cancer. LRD will limit workers' physical work, reduce work efficiency. It is gratifying that health monitoring plays an essential role in the prevention and treatment of occupational diseases. Therefore, it is of great practical significance for workers themselves and enterprises to monitor the health of construction workers and predict the risk of LRD in construction workers.

At present, nomogram has been widely used in clinical on lung diseases (29–32), but the occupational exposure and unhealthy lifestyle are rarely involved in the previous researches. In our study, this information was collected, and finally we identified four characteristics that can predict LRD for construction workers, including years for exposure to dust, labor intensity, work in shifts, and symptoms.

One momentous predictor of occupational factors associated with LRD was years for exposure to dust. The risk of LRD increases with the increase of working years exposed to dust. That is, the more accumulated dust exposure, the higher risk of diseases, which are in line with previous studies at home (33) and abroad (34). The damage of dust exposure to the lungs is obvious and clear, robust support exists for occupational dust exposure causing adverse lung changes (35–37). The human body's ability to remove dust would be weaken due to long-term dust inhalation, resulting in a large amount of dust deposition in body, and then the lung tissue would be damaged (38). Moreover, autoimmune function of body would decline with age, and the defense mechanism would become worse.

Labor intensity was proved to be independent predictive factors for LRD in our study. Workers with heavy labor intensity were more likely to suffer from LRD than those with light intensity. Some previous studies also suggested that labor intensity was negatively correlated with health. Zhu et al. (39) pointed that the labor intensity of agricultural production had a negative impact on the health of the elderly in rural China. In addition, lung ventilation increases with labor intensity degree. A study in a coal plant (40) found that the deposition rate of coal dust (particle size ranged from 0.3 to 10 micron) in respiratory system increased with the increase of labor intensity. Therefore, the amount of dust inhaled due to increased labor intensity would also increase under occupational exposure, and then the lungs are injured.

As another important occupational factor, shift work was also a prominent predictor of LRD. It is also considered as an independent risk factor for diabetes (41), vascular events (42), chronic diseases (43), breast cancer (44). Evidence has suggested that work in shifts may disrupts the normal sleep-wake cycle, leading to insufficient sleep time and excessive fatigue (45). In the absence of sleep, workers are more susceptible to infections, which in turn develop LRD, due to decreased immunity. Consistent with prior research findings, clinical symptoms are supposed to one of the hazard factors for LRD (46–48). Symptoms are portents and manifestations of disease, major symptoms are also included in the definition of many LRD, so it can be applied to predict diseases.

Nomogram can provide the most accurate predictions by a simple graphical presentation. The nomogram showed that the combination of symptoms, years of exposure to dust, work in shifts, labor intensity had a good predictive ability for the risk of LRD among construction workers. By using the nomogram recommended in our study, occupational physicians could make an individualized assessment for potential for LRD among construction workers. With this accurate risk assessment method, professionals could identify high-risk groups and adjust health monitoring measures timely.

These findings also reminded us that construction workers should pay attention to their own lung health. To reduce the risk for LRD, more targeted and effective preventive measures should be taken in time. It is necessary to improve the wearing rate of dust masks during their work for construction workers. Moreover, employers should arrange working and resting time reasonably to reduce workers' labor intensity. Shift schedules should minimize sleep deprivation and day and night interruptions. Equally vital was that, when the construction workers expressed obvious symptoms, they should be timely arranged for medical screening and transferred from the dust exposure post.

However, there were still some deficiencies in this study. First of all, due to limitation of sample sizes, we combined all types of LRD. In future, we will continue to collect samples to obtain sufficient quantity of a certain disease and improve the accuracy of analysis. At the same time, the newly collected data would be used as an external validation to evaluate the nomogram model. Second, the degree of labor intensity measured by three questions is rough. Next we will consider using the energy metabolic rate and heart rate to judge the degree of labor intensity. Finally, due to the inherent design defects of the cross-section study, causal relationship cannot be derived. In any case, it is a meaningful exploration.

## Conclusion

In summary, years of exposure to dust, work in shifts, labor intensity, symptoms are hazard factors affecting the risk of LRD among construction workers. The nomogram established in this study can be used to predict the risk of LRD in construction workers and provide help for occupational physicians to adjust health monitoring measures and formulate effective preventive and intervention measures to reduce the risk of LRD.

## Data availability statement

The raw data supporting the conclusions of this article will be made available by the authors, without undue reservation.

## Ethics statement

The studies involving human participants were reviewed and approved by Wuhan Prevention and Treatment Center for Occupational Disease Ethics Committee (2022-WZF02). The patients/participants provided their written informed consent to participate in this study.

## Author contributions

XC, WY, and JieW contributed to study conception and design, contributed to drafting of the manuscript, and revision for important intellectual content. XC, YL, JJ, YY, and SW contributed to data acquisition. XC, JingW, and GL contributed to data analysis and interpretation. All authors revised the manuscript and approved the final version before submission. The corresponding author had final responsibility for the decision to submit for publication.
