# Supplementary material for: A nomogram for predicting lung-related diseases among construction workers in Wuhan, China
Source: Front Public Health. 2022 Dec 12;10:1032188. doi: 10.3389/fpubh.2022.1032188 (PMC9792134; doi:10.3389/fpubh.2022.1032188)
Supplement: Supplementary file 1 [file Data_Sheet_1.docx]

For the classification of labor intensity, please refer to the original text of "Experimental Course on Occupational Hazard Detection" (Figure 1), and the corresponding English translation version is shown in Table 1.


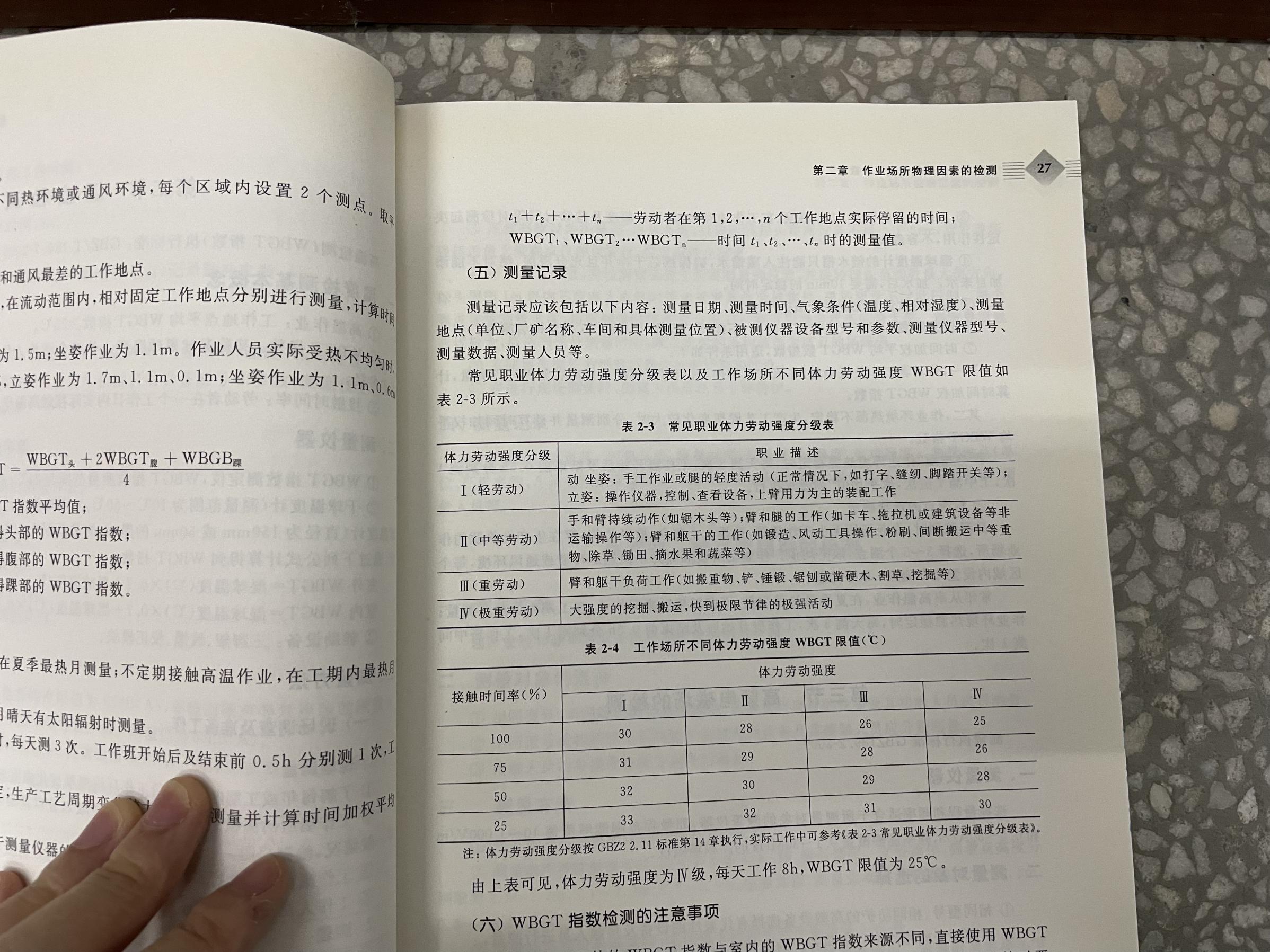


Fig 1. Common occupational labor intensity classification (Chinese)

Table 1. Common occupational labor intensity classification (English translation)

| Classification of labor intensity | Description of occupation |
| --- | --- |
| I  (light) | Sitting position: manual work or light activity of the legs (under normal conditions, typing, sewing, foot switch, etc).  Standing position: Operating the equipment, controlling and viewing the equipment, and performing the assembly work mainly with the force of the upper arm |
| II  (moderate) | Continuing work using the hands and arms (such as sawing wood). Normal work using the arms and legs (operation of non transport equipment such as trucks, tractors or construction equipment). Normal work using the arms and torso (such as forging, pneumatic tool operation, painting, intermittent handling of medium heavy objects, weeding, hoeing, picking vegetables and fruits) |
| III  (heavy) | Weight-bearing work using the arms and torso (such as heavy lifting, shovelling, hammering, sawing, planing or chipping hard wood, weeding, digging) |
| IV  (extremely heavy) | High-intensity digging or handling, and extremely strong activities that are approaching the limit rhythm |
